# The emergence of obesity in type 1 diabetes

**DOI:** 10.1038/s41366-023-01429-8

**Published:** 2023-12-14

**Authors:** Martin T. W. Kueh, Nicholas W. S. Chew, Ebaa Al-Ozairi, Carel W. le Roux

**Affiliations:** 1 UCD School of Medicine and Medical Science, UCD Conway Institute of Biomolecular and Biomedical Research, University College Dublin, Dublin, Ireland; 2 Royal College of Surgeons in Ireland & University College Dublin Malaysia Campus, Dublin, Malaysia; 3 Department of Cardiology, National University Heart Centre, National University Health System, Singapore, Singapore; 4 Dasman Diabetes Institute, Kuwait City, Kuwait; 5 Department of Medicine, College of Medicine, Jabriya, Kuwait; 6 Diabetes Complications Research Centre, University College Dublin, Dublin, Ireland

**Keywords:** Type 1 diabetes, Obesity

## Abstract

Obesity, a chronic low-grade inflammatory disease represented by multifactorial metabolic dysfunctions, is a significant global health threat for adults and children. The once-held belief that type 1 diabetes is a disease of people who are lean no longer holds. The mounting epidemiological data now establishes the connection between type 1 diabetes and the subsequent development of obesity, or vice versa. Beyond the consequences of the influx of an obesogenic environment, type 1 diabetes-specific biopsychosocial burden further exacerbates obesity. In the course of obesity management discussions, recurring challenges surfaced. The interplay between weight gain and escalating insulin dependence creates a vicious cycle from which patients struggle to break free. In the absence of weight management guidelines and regulatory approval for this population, healthcare professionals must navigate the delicate balance between benefits and risks. The gravity of this circumstance highlights the importance of bringing these topics to the forefront. In this Review, we discuss the changing trends and the biopsychosocial aspects of the intersection between type 1 diabetes and obesity. We highlight the evidence supporting the therapeutic means (i.e., exercise therapy, nutritional therapy, adjunct pharmacotherapy, and bariatric surgery) and directions for establishing a more robust and safer evidence-based approach.

## Introduction

The global prevalence of obesity has nearly tripled since 1975 with an estimated five million deaths in 2019, driven by comorbidities such as diabetes [1, 2]. Since 1980, there has been a fourfold surge in diabetes, contributing to a major cause of premature mortality [3]. In the USA, type 1 diabetes (T1D) represents about 5.6% of all cases of diabetes in adults [4]. Historically characterised as a phenotype prevalent among individuals who are lean, T1D has now been found to be influenced by factors beyond the autoimmune process [5–7]. The prevalence of T1D is projected to increase globally from 3.7 million in 2021 to ~13.5–17.4 million in 2040 [8]. The accelerated impairment of pancreatic β-cells due to obesity becomes evident amid the rapid socioeconomic and nutrition transition. In the context of type 2 diabetes (T2D), a transformative shift is unfolding in clinical care practices, primarily focused on the realisation of double-digit weight loss as a revolutionary step to address both T2D and obesity effectively [9]. However, the lack of guideline recommendations and challenges for obesity management while achieving optimal glycaemic control in patients with T1D, undermine advancements. Therefore, this Review paper aims to discuss the global burden of obesity in T1D, to provide clarity on the drivers of obesity in T1D, and to discuss the existing evidence-based knowledge of obesity management strategies in T1D.

## Search strategies

References for this Review were retrieved by searching PubMed (MEDLINE) using the search terms: “obesity”, “physical activity”, “exercise”, “nutrition”, “diet”, “obesity pharmacotherapy”, “GLP-1 receptor agonist”, “SGLT2 inhibitor”, and “bariatric surgery” in combination with “type 1 diabetes”. We included references from identified articles up to June 2023, supplemented by a manual search for relevant articles.

## Multinational patterns of obesity with type 1 diabetes

The link between childhood/youth obesity and an increased T1D incidence is ascertained [10, 11]. Higher BMI percentiles are positively associated with incident T1D among adolescents (16 to 19 years), with approximately 25% greater risk observed for each incremental standard deviation (SD) in BMI [11]. Validated with a T1D genome-wide association study (GWAS), a Mendelian randomisation study corroborated a causal role for higher childhood body size on T1D risk with an odds ratio (OR) of 1.9 (95% confidence interval CI: 1.2 to 3.1) [12]. Interestingly, the study predicted a ~ 22% reduction in T1D cases if children with severe obesity reduced their body weight by ~10%, proposing a theoretical existence of a critical window to mitigate T1D [12].

In youth with diagnosed T1D, large-scale registries from the SEARCH (USA-based), Type 1 Diabetes Exchange (USA-based), Diabetes Patienten Verlaufsdokumentation (European-based), and SWEET registry (global) have estimated the prevalence of overweight and obesity to range between 15.3% and 36.0% [13–16]. SWEET registry provided insights into the evolution of diabetes care practices among young people (<25 years old) across 22 centres from Europe, India, and Canada for 10 years [17]. The study revealed a significant improvement in the BMI-standard deviation score (SDS) from 0.6 (2008 to 2010) to 0.4 (2016 to 2018) [17]. In contrast, the DCCT study (*n* = 507, aged 8–16 years) demonstrated a relatively stable prevalence trend of overweight/obesity from 1999 (~27%) to 2009 (~31%), despite the increasing implementation of intensive insulin therapy (~52 to ~97%) [18]. Additionally, a study in the UK (*n* = 1318, aged 2–15 years) found no linear association between T1D and BMI [19]. Considering the BMI discrepancy in the paediatric context, it is essential to assess predictors for personalised risk-factor-specific intervention strategy [20]. Sociodemographic profiles, glycaemic control, diabetes treatment, mental health, and cardiovascular risk factors have been identified to alter the obesity trajectories [20]. Using advanced dual-energy x-ray absorptiometry, a body composition meta-analysis of 24 studies found that children with T1D had a greater fat mass (kg) (mean difference MD: 1.2, 95%CI: 0.3−2.1, % difference: 9.3) and body fat % (MD: 2.3, 95%CI: 0.3−4.4, % difference: 9.0) than typical developing children [21]. Future comparative studies should assess the applicability of different obesity measurements in phenotyping obesity in T1D to ensure reliable epidemiological data.

Between 1986 and 2007, obesity prevalence among adults (>18 years of age) increased from 3.4% to 22.7%, outpacing the general population and was not due to age-related changes [22]. A USA-based study analysed National Health Interview Survey data from 2016 to 2021 and found that the prevalence of overweight and obesity among adults with T1D was ~34% and ~28%, respectively [23]. Similar proportions of overweight and obesity were seen in people without diabetes (~36% and ~28%, respectively) [23]. Parallelly, these comparable findings to the general population were mirrored in studies from Europe (Belgium, Sweden, and Austria), Korea, and Mexico [24–28]. This, however, should not obscure the concerning trend since obesity was once a seldom-seen phenomenon in this population.

## The distinctive biopsychosocial factors contributing to increased obesity in type 1 diabetes

**Fig. 1:**
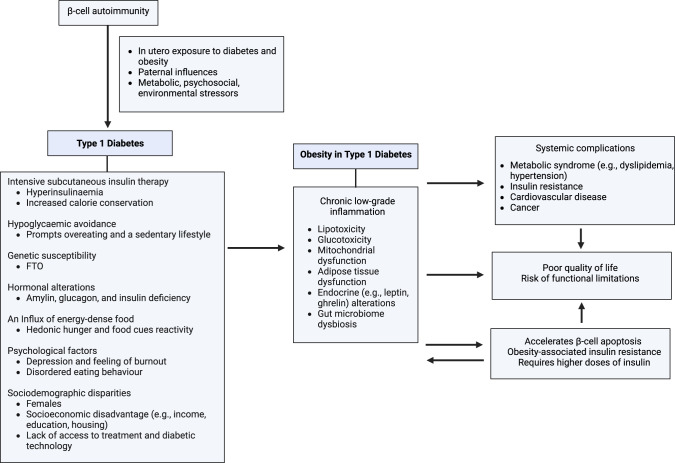
A summary of variables contributing to obesity in T1D.

The obesogenic landscape, characterised by an influx of energy-dense food and a prevailing inclination towards sedentary behaviours, is widely recognised as a driving force of the obesity surge [29]. It has also been extensively highlighted that obesity is linked to genetic, political (industry influences, suboptimal regulation), socioeconomic (e.g., disparities, food insecurity) and cultural (e.g., stigma, lack of support) factors [29]. Variables contributing to obesity in T1D are summarised in Fig. 1. The unique challenges faced by people with T1D, as detailed in the following paragraphs, have not received sufficient attention.

First, there remains an inadequate comprehension of obesity in the pathogenesis of T1D, which may impede effective prevention and treatment strategies. The development of T1D follows distinctive pathways, distinguished by the interplay of the autoimmune process and the extrinsic stressors on insulin demand [5, 6]. Our understanding of this connection largely hinges on the ‘accelerator hypothesis’ proposed in 2001, which postulated a shared pathogenesis of insulin resistance underlying the emergence of both T1D and T2D during childhood [5]. This theory continues to be argued against and affirmed in the past two decades. A Polish retrospective study (*n* = 559, aged <14 years) challenged the idea that increasing obesity rates explain the rising incidence of T1D, as it did not observe a corresponding increase in BMI among diagnosed children over time [30]. However, another study in Poland found that high BMI at T1D onset accelerated β-cell depletion and elevated inflammatory cytokines, irrespective of C-peptide level [31]. In preclinical research, a high-fat diet-induced mouse model of non-immune diabetes demonstrated that diabetes can occur via β-cell fragility [32]. This fragility can lead to the occurrence of glucotoxicity and lipotoxicity within the islets, contributing to the pathophysiology of the disease [5, 32].

Multi-omics data have expanded our views on overlapping molecular signatures. Through metabolomic investigations, it has been elucidated that intersecting metabolite pathways exist between T1D and T2D, evidenced by the upregulation of branched-chain and aromatic amino acids, glutamine cycle, glycolysis, and triglyceride metabolism [33]. Fat mass and obesity-associated (FTO) gene is implicated in predicting obesity in T1D, suggesting its potential role in genetic testing [7]. Among non-autoimmune T1D (type 1b) patients, a GWAS study identified thirteen novel loci, with nine linked to obesity [34]. Alpha-1,2-glucosyltransferase (ALG10), calneuron 1 (CALN1), EPH receptor B4 (EPHB4), nuclear factor IB (NFIB), and thioredoxin (TXN) were among the genes that represented these loci [34]. It will be interesting to examine different cohorts for potential convergences in autoimmune (i.e., type 1a) diabetes. A meta-analysis of 21 studies indicated that first-degree relatives with diabetes or obesity were predisposed to a higher risk of childhood-onset T1D. Compared to normal maternal weight, maternal with obesity increased the risk of childhood-onset T1D with a relative risk (RR) of 1.3 [35]. Notably, concerning diabetic status, the effect of maternal T1D (RR = 4.5) on childhood-onset T1D risk was the greatest, followed by gestational diabetes mellitus (RR = 1.7) [35]. Interestingly, paternal with T1D carried a 1.5-fold higher risk of transmitting T1D to children compared to maternal influence [35]. This disparity of parental influence warrants further understanding, particularly in the genetic role of human leukocyte antigen and insulin resistance. An analysis of the TEENDIAB cohort (*n* = 610) found that offspring with maternal T1D led to two-fold higher odds of abdominal obesity in children than in mothers without diabetes [36]. Metabolomics profile, however, could not explain the causal link [36]. Nevertheless, a dual association was identified where maternal T1D correlated with higher obesity risk in children, and vice versa [35, 36]. Overall, it is clear that there exists a relationship between T1D and obesity/T2D; whether the metabolic dysfunction is a consequence of autoimmunity or vice versa requires unravelling. Bridging the gap between omics associations and their functional relevance can propel our mechanistic understanding of the accelerator hypothesis.

Second, treating obesity is complicated by intensive insulin therapy, the standard of care for T1D, which paradoxically causes weight gain, creating a challenging dilemma for achieving weight management goals [37]. Such a phenomenon involves a dynamic interplay between exogenous insulin-related physiological changes (such as an imbalance between peripheral and hepatic insulin distribution, and calorie conservation) and psychological adaptation to avoid hypoglycaemia [6, 22, 38]. Hypoglycaemic fear is an additional hurdle for people living with T1D to exercise, leading to a significant proportion of adults with T1D not fulfilling the recommended levels [39]. The same worry prompts excessive carbohydrate-centric consumption. The findings from a longitudinal analysis (*n* = 600) showed that participants with T1D demonstrated a higher mean ultra-processed food intake of 7.6 servings/day (vs. 6.6 in the control group; *p* < 0.01) at baseline and of 5.6 servings/day (*vs*. 4.6 in the control group; *p* < 0.01) at 14-year follow-up [40]. Additionally, insufficient nutritional counselling on healthy eating practices makes compliance challenging [41]. Compared to peers, adolescents with T1D had higher rates of disordered eating behaviour and eating disorders than peers [42]. Long-term commitment and ongoing distress towards hypoglycaemia may lead to struggles in maintaining a healthy relationship with food. Consequently, the development of obesity leads to insulin resistance, which necessitates greater amounts of insulin. This creates a challenging situation, as the escalated insulin doses can further worsen weight gain [6].

Third, health disparities in T1D lead to a wide variation in disability-adjusted life years (DALY), with unaddressed gaps [43]. Social patterning of T1D can impact obesity or vice versa through differential vulnerability and differential exposure [44, 45]. A Swedish study (*n* = 16,365, age ≤22) revealed that a higher BMI acts as a mediator, linking lower maternal education to an elevated risk of developing T1D [46]. The finding concurs with the differential vulnerability explanation for social disparities, where unawareness can leave children more prone to unhealthy influences and augmented psychosocial stress. Other differential vulnerabilities include females, low household income, and lack of access to insulin therapies and diabetes technology [14, 43]. Without consistent treatment access, this may potentially accelerate β-cell apoptosis and diabetes progression. The impact of differential exposures, such as excessive carbohydrate consumption and low physical activity, on obesity in individuals with T1D has also been extensively examined [39, 40]. However, these existing studies have predominantly focused on evaluating these factors individually, which may restrict explanatory capacity in identifying the upstream differential exposures of obesity in T1D. As such, the integration of different components of risk factors (i.e., metabolic, behavioural, sociodemographic) in clinical and epidemiological research is warranted. Therefore, improved clarity on health disparities leading to obesity in T1D is essential for informed resource allocation and prioritisation.

T1D itself induces β-cell inflammation [7, 47]. Obesity further triggers a series of physiological events, including lipotoxicity, glucotoxicity, mitochondrial dysfunction, adipose tissue dysfunction, endocrine alteration, and gut microbiome imbalance, establishing a chronic low-grade inflammatory state [7, 47]. Consequently, this dual burden of T1D and obesity leads to systemic complications, such as cardiovascular diseases (CVD), cancers, and metabolic syndromes related to insulin resistance [6, 48]. Recognising the profound biopsychosocial impacts, calling for action on obesity care is critical at present.

## Treatments for obesity in patients with type 1 diabetes

### Exercise therapy

Exercise is a crucial pillar for weight loss, but a heavier preparatory load, especially in T1D, often exhausts this effort. A comprehensive overview of exercise in T1D has previously been reviewed [49, 50]. Simply classifying exercise efforts into aerobic, anaerobic, or mixed aerobic/anaerobic may not reflect the nuanced interplay of the energy system [49, 50]. To bypass repetition, we discuss exercise modalities focusing mainly on endurance, explosive, resistance, and high-intensity intermittent (HIIT) exercises.

Endurance exercise (e.g., running or cycling) intensity can be characterised by metabolic equivalents (METs), which are unit measurements to quantify energy used during exercise, with one MET equivalent to oxygen consumed at rest (~3.5 mL of oxygen per kilogram of body mass per minute) [51]. Three groupings have been classified: light (1.5–2.9 METs), moderate (3.0−5.9 METs) or vigorous (>6 METs) [50]. Light-to-moderate intensity increases post-exercise hypoglycaemia risk, while >45 min of moderate-to-vigorous intensity increases nocturnal hypoglycaemia risk [50]. Achieving a pre-exercise glucose recommended range of 145 mg/dL through carbohydrate feed is a more optimal approach than reducing insulin dose reduction [50]. On the other hand, explosive exercise (e.g., sprinting) is anaerobic-based and promotes hyperglycaemia [50]. Its integration into pre- and post-endurance exercises can counterbalance hypoglycaemia risk [50].

Resistance exercise (e.g., weightlifting) slightly raises glycaemia, especially in the morning, but this effect diminishes with heavier loads and less repetition [49, 50]. Given its inherent exercise mode to reduce glucose fluctuation, performing resistance exercise before endurance exercise may mitigate hypoglycaemia risk [49, 50]. A systematic review meta-analysis of 14 randomised controlled trials (RCT) involving 509 youth with T1D evidenced that a combination of aerobic and resistance exercise yielded a more optimal health outcome regarding glycaemic level, insulin dose and cardiorespiratory fitness [52]. Similarly, HIIT (e.g., intense exercise followed by 10 seconds to 5 minutes of recovery) subtly influences the glycaemic level [50]. Nocturnal hypoglycaemia may occur when HIIT is performed late noon or insulin correction for post-exercise hyperglycaemia [50]. Caution should be exercised during HIIT as symptoms mimicking hypoglycaemia can occur despite being hyperglycaemic [50]. An advisable starting point for glucose concentration in resistance exercise and HIIT is approximately 90 mg/dL [50].

Exercise prescription requires careful consideration of many factors, including pre- and post-exercise glucose target concentration, lifestyle (work, stress, sleep patterns), medical history, and exercise type, length, and level. [53] A consensus statement has put forth strategies for exercise-related glucose excursions through insulin and carbohydrate adjustment. [49] Breakthroughs in insulin pumps, continuous glucose monitoring, and sensor-automated insulin devices help control glycaemic levels around most forms of exercise and hold the most optimistic hope in exercise safety. [54]

### Nutritional therapy

The dietary importance detailed by the American Diabetes Association for active doctor-patient collaboration underscores the significance of medical nutrition therapy (MNT) [55]. MNT emphasis in T1D receiving multiple daily injections or insulin pump therapy for carbohydrate counting resulted in a decrease of HbA1c of 1.0% to 1.9% after six months and maintained at 6.9% for 6.5 years [56]. MNT effectiveness can be sustained with education on an individualised calculation of insulin-to-carbohydrate ratios and accountability cultivation to encourage adherence [57].

There is, however, no ideal nutrition prescription for MNT. In adults with T1D, the Nutrition Practice Guideline concluded an insignificant contribution of macronutrient composition and energy intake [56]. No discernible impacts on HbA1c and cardiovascular risk factors were observed across varying carbohydrate (~39% to 57% energy) and fats (~27% to 40% energy) amounts [56]. However, lower dietary carbohydrates may attenuate blood glucose fluctuation, reducing the error rate for insulin administration [56]. Notwithstanding the mixed result on HbA1c, consuming 21 to 25 g/day (adult female) and 30 to 38 g/day (adult male) of fibres for overall metabolic health is recommended [56]. Additionally, the beneficial effects of replacing caloric sweeteners with nutritive or non-nutritive sweeteners require further elucidation [56]. Modifications to decrease the saturated to unsaturated fats ratio showed little influence on glycaemic levels, despite a favourable lipid profile [56].

A systematic review was conducted to highlight the MNT effectiveness among adolescents with T1D [58]. The findings aligned with the MNT evidence in adults reporting mixed glycaemic levels and negligible correlation with BMI [58]. Nevertheless, MNT should still be implemented and viewed within a holistic overall lifestyle intervention, combined with physical activity [56, 58]. On examination of dietary intake of patients with T1D, lower overall energy intake was observed with sufficient protein intake [59]. However, diets tend to fall short in aspects of fat, carbohydrate, fibre, and micronutrients [59].

The adoption of dietary patterns is increasingly favoured as a means to maintain sustainable nutritional composition. In a one-year real-life experience, a eucaloric, very low-carbohydrate diet (carbohydrate <50 g/day) significantly improved glycaemic levels and severe hypoglycaemia (30.3% to 0% after diet initiation), with no instances of diabetic ketoacidosis (DKA) [60]. The adoption of a low carbohydrate regimen mandates a concomitant reduction in insulin dose [60]. As such, hypoglycaemia risk can be mitigated, and the inherent low carbohydrate composition serves as a mechanistic safeguard from hyperglycaemia [60]. Caution should be exercised in interpreting the results due to the limited sample size (*n* = 33) and only highly motivated individuals included [60]. Another 3-month pilot RCT study of adults aged 19−30 years with T1D (*n* = 38) assessed the effects of a low carbohydrate diet (<14% calories), Look AHEAD diet (<30% calories from fat and <10% fat from saturated fat) and Mediterranean diet on weight and glycaemic outcomes [61]. The findings indicated that there were no superior co-weight and glucose benefits with caloric restriction, suggesting the key determinant of effectiveness may be patient preference and adherence [61]. Nevertheless, the safety of each diet needs to be evaluated. The impending extension report (third to ninth months) on re-randomisation conducted during COVID-19 period may entail the outcome of a more tailored diet plan, and further inform the implications of telemedicine in dietary interventions.

### Adjunct pharmacotherapy

Adjuncts to insulin can address unmet needs of reducing weight and complications among patients with T1D and obesity. Pramlintide, a synthetic analogue of human amylin, currently represents the sole approved adjuvant therapy for T1D in the USA [62]. Its co-administration with insulin has improved long-term glycaemic control and weight loss [63]. New amylin analogues are currently in the pipelines. The findings from a phase 2 clinical trial evaluating the efficacy of XP-3924, a novel fixed-ratio co-formulation of pramlintide and regular insulin, have reported substantial glycaemic variability improvements, potentially substituting regular insulin in the forthcoming time [64].

Glucagon-like peptide-1 receptor agonists (GLP1-RA) and sodium-glucose cotransporter-2 (SGLT2) inhibitors have mounted preferences because of their beneficial effects on glycaemic control, weight loss, and overall cardiometabolic parameters [65, 66]. Adjunct GLP1-RA and SGLT2 inhibitors consistently presented with reduced mean effects on body weight and HbA1c [6]. An overview of ongoing, recruiting, and upcoming trials of adjunct pharmacotherapies with potential for obesity management is summarised in Table 1.

**Table 1:** An overview of ongoing, recruiting, and upcoming trials of anti-obesity medications as adjunct therapies in T1D.

Adjunct medication	ClinicalTrials.gov identifier	Phase(s)	Age inclusion (years)	Estimated sample size	BMI inclusion (kg/m^2^)	Other intervention(s)	Placebo-controlled	Primary outcome	
								Measure(s)	Timeframe
GLP1-RA									
GLP1	NCT04355832^b^	1	18−50	40	<40	/	✓	Catecholamines levels	3 years
Liraglutide	NCT02516657^c^	3	15−21	5	NR	/		Mean weekly blood glucose	2 weeks
Liraglutide	NCT03011021^b^	1 and 2	≥18	40	NR	Umbilical cord blood-T regulatory cells infusion [biological]		Adverse events and signs of toxicity	2 years
Liraglutide	NCT05794581^b^	1	18−65	24	25−35	CT-868 [dual GLP1 and GIP receptor modulator]	✓	Area under the curve in glucose metabolism	Up to 4 days
Liraglutide	NCT03182426^c^	1 and 2	18−45	60	<35	Plerixafor, Alemtuzumab Anakinra Etanercept		C-peptide area under curve and serious adverse event rate	3, 6, 9, 12, 18 and 24 months
Semaglutide	NCT05537233^b^	2	18−60	80	≥30		✓	Continuous glucose monitoring-measured time in range >70% with time below range of <4% and reduction in body weight by 5%	26 weeks
Semaglutide	NCT05819138^b^	3	18−40	60	20−35		✓	Central and peripheral arterial stiffness	4 weeks
Semaglutide	NCT05822609^a^	2	≥18	60	NR		✓	Kidney cortical relaxation rate	26 weeks
Semaglutide	NCT05205928^b^	2 and 3	≥18	28	≥22	Closed-loop insulin system	✓	Percentage of time of plasma glucose levels spent in target range (3.9 to 10.0 mmol/L)	4 weeks
Dulaglutide	NCT05478707^a^	2	18−40	64	19−27	Exercise training	✓	Microvascular blood volume	14 weeks
SGLT2 inhibitor									
Sotagliflozin	NCT05696366^a^	1 and 2	18−70	22	18.5−35	Volagidemab	✓	HbA1c changes	12 weeks
Dapagliflozin	NCT05541484^b^	4	18−75	20	NR	Biosense breath ketone analyser		Blood and breath ketone levels	4 weeks
Dapagliflozin	NCT04333823^b^	3	12−18	100	Within 99.9th percentile	/	✓	Glomerular filtration rate	16 weeks
Dapagliflozin	NCT04049110^b^	3	18−65	24	20−29	/		Mean amplitude of Glucose Excursions upon exercise	From completion of physical exercise at day 7 of each intervention period to 72 h after
Dapagliflozin	NCT03878459^b^	4	≥18	120	NR	Pioglitazone,	✓	HbA1c change	28 weeks
Dapagliflozin	NCT03704818^c^	1	18−70	22	18.5−35.0	/	✓	Counterregulatory response to hypoglycaemia	12 weeks
GLP1-RA + SGLT2 inhibitor									
Liraglutide, semaglutide, dapagliflozin	NCT05390307^a^	N/A	21−65	60	≥25	Intensive nutrition, bariatric surgery, and usual care		Weight change	26 weeks
Semaglutide, dapagliflozin	NCT03899402^b^	2 and 3	18−75	114	≥25	Insulin	✓	HbA1c change	6 months

A search was conducted on ClinicalTrials.gov on June 21, 2023 to identify registered trials.

*GIP* glucose-dependent insulinotropic polypeptide, *GLP1-RA* glucagon-like peptide-1 receptor agonists, *SGLT2* sodium-glucose cotransporter-2.

*NR* not reported, *N/A* non-applicable.

^a^Not yet recruiting.

^b^Recruiting.

^c^Active, not recruiting.

#### Glucagon-like peptide-1 receptor agonists

##### Efficacy

Adjunct GLP1-RA suppresses glucagon release and delays gastric emptying, to counterbalance weight gain through a reduction in prandial insulin dosing [67]. In a pooled analysis of 2609 patients across eight RCTs, adjunct liraglutide reduced body weight by 4.0 kg (95%CI: −4.5 to −3.4) compared to placebo [68]. According to a post hoc analysis of ADJUNCT ONE and ADJUNCT TWO trials, the greatest placebo-adjusted HbA1c (−0.3% and −0.4%), body weight (−5.0 kg and −4.8 kg), and daily insulin dose (~−12% and ~−10%) were observed after 26 weeks with adjunct liraglutide 1.8 mg [69]. While the reductions in HbA1c, body weight, and daily insulin dosage in patients treated with liraglutide (0.6 mg, 1.2 mg, 1.8 mg) were unaffected by baseline HbA1c, BMI, and insulin regimen subgroups, the authors pointed out that residual β-cell function might be of greater relevance in this context [69]. This idea had previously been translated in the context of T2D – a 3-year phase 3 SCALE study [70]. This RCT, involving a cohort of 2254 patients with overweight or obesity and pre-T2D, showed that once-daily subcutaneous liraglutide 3.0 mg led to a higher odd of >15% weight reduction (OR 4.0; 95%CI 2.6−6.3) [70]. Remarkably, by week 160, the regression from prediabetes to normoglycaemia was 65.8% in the liraglutide group (vs. 36.3% in the placebo group) [70]. A parallel investigation on GLP1-RA early obesity-related mechanistic rewiring may pave the way for a more optimised therapeutic prospect in T1D. Investigating the potential to mitigate or reverse early β-cell impairments, simultaneously provides an opportunity to revisit the accelerator hypothesis.

A secondary outcome analysis of the Lira pump trial (*n* = 44) reported that liraglutide 1.8 mg lowered fat mass [71]. Concerningly, it was accompanied by a reduction in lean mass (−2.5 kg vs. 0 kg in the placebo group; *p* < 0.001) [71]. This finding should be interpreted with caution as there was no emphasis on dietary guidance provided in the study [71]. Additional studies are needed to gain insights into the mediating role of adjunct low-caloric diet. Thrice daily adjunct short-acting and weekly subcutaneous extended-release exenatide resulted in weight reduction, but the results for achieving HbA1c so far have been unsatisfactory [72, 73].

##### Safety and tolerability

The pooled analysis of RCTs investigating the effects of GLP1-RA on T1D revealed a higher incidence of gastrointestinal disorders in the liraglutide group with an OR of 3.0 [68]. There were no significant differences between the liraglutide group and placebo group concerning DKA, hypoglycaemia and severe hypoglycaemia [68]. In a phase 3 ADJUNCT-ONE trial, it was observed that there was a dose-dependent increase in hyperglycaemia with ketosis ranging from 0.6 mg to 1.8 mg [74]. This increase corresponded to a dose-dependent increase in nausea and an increase in the reduction of insulin dose [74]. Conversely, the generally well-tolerated nature of liraglutide 3.0 mg observed in the pre-T2D SCALE trial [70] lends support to its potential safety in the early phase of T1D, particularly in cases where residual β-cell function remains. The higher prevalence of T1D in the paediatric group raises concerns regarding efficacy and tolerability. Additionally, the cost-effectiveness of this approach should also be weighed against lifestyle modifications.

##### Effectiveness in real-world studies

A real-world study of adjunct liraglutide (*n* = 11) conducted a decade ago reported a reduced daily insulin dose (−19.2%), HbA1c (−0.4%), and body weight (−3.0 kg) after 10 weeks [75]. More recently, a 52-week study (*n* = 76) yielded comparable results for daily insulin dose and HbA1c but indicated a more substantial reduction in body weight (−5.1 kg) [76]. In another study, combining both GLP1-RA and SGLT2 inhibitor resulted in the greatest percentage of weight loss (9.0%) compared to a single prescription, after 12 months [77]. This combination improved HbA1c, total cholesterol, and LDL-cholesterol while remaining safe from DKA and hypoglycaemia [77]. Notably, the implementation of a structured risk prevention programme and sick-day guidance were applied to all enroled patients, [77] highlighting the importance of investing in patient education and the provision of adaptable care.

#### Sodium-glucose cotransporter-2 inhibitors

##### Efficacy

Sotagliflozin, a dual SGLT1 and SGLT2 inhibitor, was investigated in the Tandem phase 3 clinical trials [78, 79]. A post hoc analysis of Tandem 1 and 2 trials (mean age 43.6; SD: 13.5) examined the efficacy and safety of sotagliflozin in T1D after a 6-week insulin optimisation period [80]. Both the sotagliflozin 200 mg and 400 mg groups exhibited weight reduction, with placebo-adjusted decrease of 3.2% and 4.2% respectively, after 52 weeks [80]. 22.7% and 30.7% in the sotagliflozin 200 mg and 400 mg groups lost >5% body weight. [80] The DEXA scan showed that the reduction in fat mass was primarily responsible for the change, as a result of calorie loss, a decrease in insulin dose and ketogenesis [80]. Similar results of weight loss while achieving HbA1c goals were reported in a 12-week trial with younger adults (18−30 years old) [81]. The heightened emphasis on cardiovascular data has driven an expansion of cardiovascular outcome trials in the scope of T2D [82]. However, such data remained unanswered in T1D. A modelling study of Tandem 1 to 3 trials demonstrated a significant decrease in CVD and kidney failure risk scores, estimated at 6.5% and 5.0% respectively, at week 24 [83]. Prospective RCT, especially in people at high risk of cardiovascular and/or kidney disease, is needed to understand benefit-to-risk ratio regarding the impact of intentional weight loss on cardiorenal outcomes.

Dapagliflozin, the first-in-class SGLT2 inhibitor, was the subject of several trials, however, no conclusive advice on the optimal dose can be drawn [84]. A network meta-analysis of 13 RCTs (*n* = 10,701) highlighted that treatment with dapagliflozin 5 mg and 10 mg decreased body weight from the baseline at MD: −3.2% (95%CI: −3.5 to −2.9%) and MD: −4.2% (95%CI: −4.6 to −3.9%) [84]. Maximum HbA1c efficacy of dapagliflozin was estimated to be −6.2% at week 9, irrespective of dapagliflozin doses. After that, a rebound effect was observed, with lower efficacy if the drug continued for 6 to 12 months [85]. Nonetheless, it has been modelled that the achievement of sustained weight loss with dapagliflozin necessitates at least 42 weeks in T1D [86]. This highlights the significance of persistent adherence to weight loss goals. A DEPICT post-hoc analysis assessing renal function revealed significant changes in urinary albumin-to-creatinine ratio compared to placebo after 52 weeks of treatment [87]. Dapagliflozin 5 mg showed a reduction of 13.3%, while dapagliflozin 10 mg exhibited a more pronounced decrease of 31.1% [87]. Preclinical T1D research showed that dapagliflozin can rewire atherosclerotic properties and attenuate cardiac inflammation and fibrosis [88]. The critical next phase involves translating these cardiorenal-protective findings into adequately powered trials with prespecified endpoints.

Empagliflozin is another SGLT2 inhibitor which has been approved for T2D, however, its indication is limited in T1D given the paucity of trials. Based on the EASE clinical trials, a meta-analysis found a dose-dependent mean difference in weight loss for adjunct empagliflozin 2.5 mg (−1.5 kg), 10 mg (−2.8 kg) and 25 mg (−3.1 kg) [89]. In the EMPA-KIDNEY trial, which aimed to assess the effects of empagliflozin on renal protection, reported substantial reductions (28%) in the progression of kidney disease and cardiovascular mortality, compared to placebo [90]. A notable aspect of this trial is its inclusion of participants diagnosed with T2D and T1D, demonstrating efficacy irrespective of diabetes status [90]. Despite the minor proportion of only 1.0% (*n* = 68) patients with T1D included in this RCT, this should encourage attention to trials with similar endpoints in dedicated T1D [90]. Another timely approach is optimising its use with automatic insulin delivery. A randomised crossover trial of 24 adults found that high-dose empagliflozin (25 mg) add-on to closed-loop automated insulin delivery and sensor-augmented pump therapy significantly increased the time spent in the glucose target range (7.2% and 11.4%, respectively) after four weeks, compared to placebo [91]. Similarly, at lower empagliflozin doses of 2.5 mg and 5 mg with a closed-loop system, a 14-day RCT showed an 11 to 13 percentage point increase time in range compared to placebo [92]. These collective data represent a promising avenue of complementing obesity management for better glycaemic control and early risk recognition.

##### Safety and tolerability

DKA, a life-threatening complication, has been longstanding with far-reaching consequences on mortality and economic implications in T1D [93]. The introduction of SGLT2 inhibitors has brought about heightened apprehensions surrounding the increased risk of DKA, which can occur even in the absence of pronounced hyperglycaemia [94]. Consistent across landmark clinical trials, for every 100 patients with T1D treated with SGLT2 inhibitors, an estimated four incidences of DKA were predicted to occur annually [94]. The distribution of DKA risk In the population is not uniform, with four independent determinants accounting for the variation: BMI of more than 27 kg/m^2^, an estimated glucose disposal rate of less than 8.3 mg/kg/min, suggestive of insulin resistance, an increased total insulin dose reduction-to-baseline insulin sensitivity ratio, and dehydration [95].

By synthesising data from 15 RCTs, a meta-analysis concluded that there were no significant differences in terms of hypoglycaemia, severe hypoglycaemia, and urinary tract infections [96]. However, when compared to the placebo group, a significant association was observed between the duration of SGLT2 inhibitor treatment and a heightened risk of genital tract infection, with rates of 4.1 at 24 to 26 weeks and 4.4 at 52 weeks [96].

##### Effectiveness in real-world studies

Multinational real-world SGLT2 inhibitors evidence is beginning to take shape. In Spain and Belgium, adults (*n* = 199) with T1D showed significant reductions in HbA1c (−0.5%), weight (−2.9 kg), and daily insulin (−8.5%) after 52 weeks of SGLT2 inhibitors [97]. In a similar period, a single institution in the US observed significant reductions in HbA1c (−0.6%), weight (−1.7 kg), and daily insulin (−0.02%) in 39 prescribed patients [76]. Nationwide observational studies in Saudi Arabia, Japan, Germany, Austria, and Switzerland have all demonstrated similar outcomes about SGLT2 inhibitors’ potential in clinical practice [98–100]. Real-world data has yielded valuable insights into effective strategies for DKA mitigation. It is a reasonable prediction that DKA occurrence (10.8%−12.8%) [76, 100] is more frequent in uncontrolled clinical environments compared to clinical trials. With appropriate patient selection and intense surveillance (e.g., education on risks, prevention, and home ketone monitoring), DKA occurrence reduces from 0% to 3.5% [97, 98]. Nevertheless, a cautious approach remains imperative when integrating into routine clinical care. In chronic kidney disease, prescription of SGLT2 inhibitors is not recommended for patients with very low glomerular filtration rate [101]. We envision a mirrored risk-stratification strategy in T1D.

### Bariatric surgery

#### Effectiveness

Bariatric surgery has emerged as a viable treatment option for obesity in adults with T2D and a BMI > 35 kg/m^2^ [102]. Regarding T1D, a systematic review and meta-analysis of 30 studies (*n* = 706) with a mean age of 40.0 years and a mean BMI of 40.9 kg/m^2^ showed excess weight loss reduced by 74.6% (60.0−90.5%) after six months [103]. Contrarily, a long-term study showed weight regain from a nadir BMI of 27.5 kg/m^2^ to 30.1 kg/m^2^ after 3.5 years [104]. Additionally, the amount of insulin needed per day decreased from a mean of 92.3 IU/day pre-operatively to a mean of 35.8 IU/day post-operatively [103]. This is possibly due to an improvement in increased hepatic insulin sensitivity, and β-cell function and mass preservation [105]. Nevertheless, a long-term sustained decrease in insulin dose may still be challenging to quantify and is likely to be complicated by extrinsic influences, such as compliance with follow-up and lifestyle choices. Furthermore, glycaemic control following bariatric surgery fell short of the desired outcome considerably [103, 105]. In light of the recognised shortfalls, it is apparent that an improved clinical stratification is required to determine the optimal timing of bariatric surgery and accurately diagnose T1D from latent autoimmune diabetes in adults [106].

Commonly performed surgical procedures include Roux-en-Y Gastric Bypass (RYGB) and sleeve gastrectomy [103]. RYGB is favoured owing to the quick passage of digestive contents to the distal ileum, while bypassing the proximal small intestine, thereby boosting the release of incretin hormones (i.e., GLP1) [103, 107]. Hypertension, dyslipidemia, obstructive sleep apnoea, and microalbuminuria improved with reductions of 42.8, 25.0, 66.0, and 25.0%, respectively [108]. HDL-cholesterol increased by 13.5 mg/dl, whereas systolic blood pressure, diastolic blood pressure, LDL-cholesterol, and triglyceride improved by 10.1 mmHg, 6.2 mmHg, 9.5 mg/dl, and 11.0 mg/dl, respectively [109].

#### Safety and tolerability

With average perioperative mortality below 0.3%, bariatric surgery is deemed safe but not without risk [106]. Several common short-term problems, such as marginal ulcers, incisional hernias, oesophageal dysmotility, prolonged nausea, and nutritional deficits, were experienced by a range of 4.0%−25.0% of patients with T1D who underwent bariatric surgery [103, 110, 111]. The risks for hypoglycaemia and DKA episodes are major concerns, with each risk increasing by up to 28.6% [112] and 25.0% [113] respectively. The predisposing factors for DKA include surgical stress, suboptimal care, sudden halt or non-compliance with insulin therapy, infection, and electrolyte imbalance [106, 113]. These potentially fatal consequences highlight the importance of a multidisciplinary team in post-operative diabetes care, particularly in careful adjustment of insulin doses and consistent monitoring of blood glucose levels [103, 106].

## Future directions for obesity management in type 1 diabetes

Recent years have seen significant progress in prioritising weight loss management in patients with T1D and obesity, with few important considerations to further advance this field.

First, the growing gaps in life expectancy between patients with T1D and the general population [43] translate into a clear and pressing message for policymakers and regulators. Given concerns over safety and tolerability, anti-obesity pharmacotherapies for T1D remain classified as off-label. Recent real-world data on these off-label pharmacotherapies complement the benefits of RCT, offering a retrospective lens to assess the pharmacological effectiveness. The convergence of real-world studies with RCT findings lends credibility to external validity. In terms of safety control, appropriate preventive measures have been demonstrated to mitigate the incidence of DKA and hypoglycaemic episodes in 12 months (Table 2) [74, 76–79, 97, 98, 114–116]. This strategic amalgamation of both data present promising prospects in strengthening the rationale for drug approval and label expansion. Recognising T2D and obesity as two closely intertwined chronic diseases, Lingvay et al. advocated for the implementation of an approval pathway that transcends a simplistic binary approach [9]. At the current juncture, given the time-critical nature of β-cell apoptosis in T1D, there is a pressing need for transparency in the approval decision-making process, with patients positioned at the core of the discourse. Considering the life-threatening DKA episodes, it is equally important to ensure pharmaceutical caution superseding acceleration. A consensus from Danne et al. presented important implications for DKA management in patients with T1D treated with SGLT2 inhibitors [117]. These recommendations provide the fundamental framework for stringent risk stratification within guidelines, contributing to responsive and adaptable pharmaceutical prescriptions.

**Table 2:** An overview of the reported preventive measures and incidence rates of DKA and severe hypoglycaemia over a 12-month period.

Trial	Adjunct drug and route	Preventive measures	Doses	DKA (%)	Severe Hypoglycaemia (%)
DEPICT-1 [114], DEPICT-2 [115]	Dapagliflozin; oral once daily	- Blood glucose monitoring, min. 4 times/ day, and 6 times/day for specific intense phase. - Home blood ketone measurement. - Diet and exercise counselling. - Instructions on DKA risk. - Insulin dose adjustment (not >20%) throughout study.	5 mg	4.0; 4.1	10.5; 8.9
			10 mg	3.4; 3.7	8.4; 9.6
			Placebo	1.9; 0.4	11.5; 8.5
inTandem1 [78]; inTandem2 [79]	Sotagliflozin; oral once daily	- Prompt and systematic address of ketosis, - Instructions on DKA risk, symptoms, management - Blood/urine ketone measurement, insulin dose adjustment	200 mg	3.4; 2,3	6.5; 5.0
			400 mg	4.2; 3.4	6.5; 2.3
			Placebo	0.4; 0	9.7; 5.0
Phase III study [116]	Ipragliflozin; oral once daily	- Blood glucose monitoring – 7 times/day for specific intense phase - Insulin and adjunct ipragliflozin dose adjustment throughout study	50 mg & 100 mg	0	0
ADJUNCT-ONE [74]	Liraglutide; subcutaneously once daily	- Begin with a low dose - Gradually titrate up - Insulin dose adjustment (~25% on randomisation day, ~10% on dose escalation)	0.6 mg	0.9	9.1
			1.2 mg	0.3	6.3
			1.8 mg	0.8	8.1
			Placebo	0	10.9
Real-world study	Adjunct drug	Preventive measures	Doses	DKA (%)	Severe Hypoglycaemia (%)
Palanca et al. [97]	a. Empagliflozin b. Dapagliflozin c. Canagliflozin	- Instructions on DKA risk, symptoms, management - Home blood/ urine ketone measurement - Card describing the STICH protocol	a. 5 mg, b. 5 mg, c. 50 mg	2.6	0
			a. 10, 12.5, and 25 mg b. 10 mg c. 100 mg	4.1	0
Seufert et al, 2021 [98]	a. Dapagliflozin, b. Empagliflozin	- Highly stringent patient selection - Intense surveillance over the treatment course	a. 5 to 10 mg b. 10 to 25 mg	0	3.0
Edwards et al, 2023 [76]	Liraglutide, semaglutide, dulaglutide, exenatide, albiglutide	- Preferentially choice for patients at risk of DKA	Not reported	3.9	1.3
	Empagliflozin, dapagliflozin, canagliflozin	- Not reported	Not reported	12.8	2.6
Al-Ozairi et al, 2023 [77]	Dapagliflozin	- Enroled in a structured education program - Home blood/ urine ketone measurement, CGM for > 2 months - Emphasis on sick day rules	Not reported	1.1	0
	Liraglutide			1.2	1.2
	Combination			0	0

Second, the untapped potential within medical management warrants renewed attention on optimisation. To achieve and sustain glycaemic equilibrium, it is imperative to evaluate the potential efficacy of complementary interventions. Dietary and physical activity counselling was provided to patients in the DEPICT trials [118, 119]; nonetheless, the level of implementation of these interventions was uncertain. Thus, the status as adjunctive lifestyle measures cannot be definitely established. An existing limitation, particularly evident in studies evaluating lifestyle interventions, pertains to the smaller subset of patients, which may compromise the generalisability of the findings and biases. To conduct adequately powered studies, collaborative efforts must transcend geographical and disciplinary boundaries, facilitating comprehensive informed prescription guidelines. Currently, the multinational evidence on the efficacy of adjunct pharmacotherapies is primarily derived from high-income countries, hence highlighting a gap in data representation from low- and middle-income countries.

Third, to drive progress in the landscape of medical weight management, it is essential to place importance on understanding the patient’s perspective. In contemporary discussions on anti-obesity medication, the measure of breakthrough often revolves around the percentage of weight loss achieved [120]. A 5% weight loss is considered a responsive outcome, a 5 to 10% reduction is associated with comorbidities prevention, while a >15% reduction can improve cardiovascular outcomes [120]. Among patients with T1D, achieving a weight reduction milestone may confer further enhancements in glycaemic stability, insulin dose requirement, and potentially even the recovery of β-cell function. Poor medical adherence is a concern for sustainable and safe management. Some reasons may include forgetfulness, fear of adverse effects, and suboptimal communication with providers. In structured interviews, patients with T1D highlighted their perception of real-world benefits from GLP1-RA and SGLT2 inhibitors, though the associated risk exceeded that observed in RCTs [121]. Further qualitative research is needed to identify barriers faced to devise effective safety optimisation interventions.

**Fig. 2:**
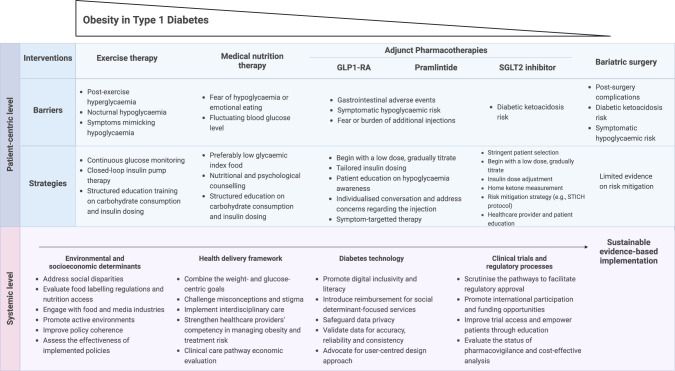
An overview of the barriers and strategies for different weight management interventions, and proposed systemic-level strategies.

Fourth, action plans on T1D-obesity treatment goals for clinicians need to be clarified. The value of a weight-centric framework in tackling adiposity-related diabetes, and glucose-centric for addressing β-cell dysfunction have been highlighted [9]. However, the coexistence of β-cell dysfunction and obesity will necessitate a novel, integrated framework that combines weight-centric and glucose-centric approaches. Having identified strategies to address individual treatment challenges (Fig. 2), [49, 117, 122, 123] a tantalizing opportunity arises to forge novel pathways in the harmonisation of treatment strategies. The subsequent step is to recognise the potential synergy between the benefits of each treatment and their ability to offset the risks of the other approach. The competence of clinicians in monitoring DKA management constitutes a critical evaluation. A national cohort in Qatar (*n* = 602 in T1D; *n* = 1011 in T2D) reported a lower admission to the intensive care unit (26.6.% vs. 38.0%), shorter hospital stays (2 days vs. 5 days), and lower inpatient mortality in T1D (1.0% vs. 7.4%) compared to T2D [124]. Correspondingly, another nationwide study in Japan (*n* = 10,442 in T1D; *n* = 13,835 in T2D) showed a lower in-hospital mortality in T1D (0.9% vs. 4.3% in T2D) [125]. The collective results reflect the clinicians’ proficiency in care, but the introduction of obesity management in T1D can complicate matters. In light of this, it should fall within the purview of trained professionals (i.e., endocrinologists and obesity physicians) to prescribe obesity management and closely monitor the patients. Furthermore, the trusted status of multidisciplinary experts as messengers highlights the critical need for curricular strategies that uphold the desired effectiveness of structured patient education.

Finally, attention to healthcare inequality should intensify. The evolving technological innovations and limited availability of off-label medications are progressively tilting towards a first-world-centric resolution. Unequal access to resources will result in disparities in glycaemic control, exacerbating obesity-related complications among socioeconomically deprived groups. Effective governance mechanisms with aligned goals are essential steps in harnessing these options to benefit the health of all individuals equitably. While the latest guidelines recommend SGLT2 inhibitors and GLP1-RA for adults with T2D and established CVD, [126, 127] an analysis of out-of-pocket costs in the USA found that, compared to the lowest quartile, individuals with the highest quartile of out-of-pocket costs were ~13% and ~20% less inclined to start GLP1-RA or SGLT2 inhibitor intervention, respectively [128]. High prescription cost and constrained reimbursement mechanisms impede widespread adoption. In T1D, these challenges are further exacerbated due to the lack of formal endorsement and approval of GLP1-RA and SGLT2 inhibitor interventions. Therefore, key societies must unify their position, addressing both present and future facets of adjunct T1D-obesity interventions. A common ground will facilitate the establishment of novel clinical care pathways and subsequent health economic evaluation.

## Conclusions

Obesity in T1D has been underrated as an emerging threat until recently. Breaking the perpetuating cycle of weight gain and increased insulin dose requirement is now a high calling. The recent work of translating clinical trials into real-world results brought upon important rationales for approval discussion and consensus-driven guidelines. Obesity management in T1D has to be underpinned by a shift in focus to a glucose-weight-centric approach with an optimal combination of adjunct interventions for an enhanced benefit-to-risk ratio. Structured education combined with individualised dosing adjustments constitutes the cornerstone for sustaining dual weight and glycaemic targets. On a systemic level, an aligned multistakeholder initiative is needed to ensure the true value of the global action plan on this T1D-obesity burden.
